# Early prediction of acute kidney injury in patients with gastrointestinal bleeding admitted to the intensive care unit based on extreme gradient boosting

**DOI:** 10.3389/fmed.2023.1221602

**Published:** 2023-08-31

**Authors:** Huanhuan Shi, Yuting Shen, Lu Li

**Affiliations:** ^1^Department of Gastroenterology, Peking University Third Hospital, Beijing, China; ^2^Department of Internal Medicine, Wuhan University of Technology Hospital, Wuhan, China

**Keywords:** XGBoost, acute kidney injury, gastrointestinal bleeding, intensive care unit, machine learning

## Abstract

**Background:**

Acute kidney injury (AKI) is a common and important complication in patients with gastrointestinal bleeding who are admitted to the intensive care unit. The present study proposes an artificial intelligence solution for acute kidney injury prediction in patients with gastrointestinal bleeding admitted to the intensive care unit.

**Methods:**

Data were collected from the eICU Collaborative Research Database (eICU-CRD) and Medical Information Mart for Intensive Care-IV (MIMIC-IV) database. The prediction model was developed using the extreme gradient boosting (XGBoost) model. The area under the receiver operating characteristic curve, accuracy, precision, area under the precision–recall curve (AUC-PR), and F1 score were used to evaluate the predictive performance of each model.

**Results:**

Logistic regression, XGBoost, and XGBoost with severity scores were used to predict acute kidney injury risk using all features. The XGBoost-based acute kidney injury predictive models including XGBoost and XGBoost+severity scores model showed greater accuracy, recall, precision AUC, AUC-PR, and F1 score compared to logistic regression.

**Conclusion:**

The XGBoost model obtained better risk prediction for acute kidney injury in patients with gastrointestinal bleeding admitted to the intensive care unit than the traditional logistic regression model, suggesting that machine learning (ML) techniques have the potential to improve the development and validation of predictive models in patients with gastrointestinal bleeding admitted to the intensive care unit.

## Introduction

Acute kidney injury (AKI) is a common morbidity with a high incidence in patients admitted to the intensive care unit (ICU). It is associated with significant mortality, and a considerable proportion of patients develop AKI that progresses to chronic kidney disease (1–3). AKI has often been reported to occur in patients with gastrointestinal bleeding (GIB), especially those admitted to the ICU due to massive blood loss, leading to renal hypoperfusion secondary to intravascular volume depletion and eventually AKI (4, 5). AKI has been reported to develop in 1–11.4% of patients with acute GIB (6, 7). A systematic review aimed to explore the incidence and mortality of renal dysfunction in cirrhotic patients with acute GIB revealed that the pooled incidence of AKI was 25% (8).

For critically ill patients with GIB concomitant with AKI, hospitalization times may be prolonged, and costs will greatly increase, bringing a heavy burden to the medical system (9–11). Approximately 20% of patients with severe GIB and new-onset AKI can restore normal renal function if appropriate and effective interventions are performed on time (12). However, the lack of early prediction tools for AKI is a major challenge for ICU clinicians. Early recognition, risk assessment, and care for AKI can improve clinical outcomes and reduce the high healthcare costs of these patients. To assist physicians with risk assessment of AKI, various prediction models have been developed across various patient populations with varying degrees of predictive accuracy.

Models being built using machine learning (ML), which are mathematical models to make decisions and predictions based on datasets, have become popular, and ML techniques have been widely used clinically for prognosis prediction, including AKI (13). ML has shown better performance and low error rates in predicting clinical outcomes compared to traditional prediction tools such as logistic regression and Cox regression analysis. Moreover, ML has been widely used clinically to predict survival (14). Extreme gradient boosting (XGBoost) is recognized as a more advanced ML algorithm with much higher prediction accuracy and operation efficiency and has been widely applied for diagnosis and prognostic prediction (15). Recently, the use of ML models for AKI prediction has been rapidly growing in different clinical settings. Yue et al. reported that the XGBoost model had the best predictive performance for AKI in critically ill patients with sepsis (16). Zhang et al. evaluated five machine learning methods including XGBoost, adaptive boosting, random forest, logistic regression, and multi-layer perception to develop AKI risk prediction models in critical care patients with acute cerebrovascular disease and found that the XGBoost model was better at predicting AKI risk in patients with acute cerebrovascular disease than other models (17). However, the efficacy of XGBoost in predicting AKI in critically ill patients with GIB remains unclear.

This study aimed to use XGBoost to construct a predictive model to evaluate AKI risk in critically ill patients with GIB and use the publicly available eICU Collaborative Research Database (eICU-CRD) as a data source for the training cohort and the Medical Information Mart for Intensive Care-IV (MIMIC-IV) database as a data source for the validation cohort. This study explored the accuracy of XGBoost for the construction of AKI prediction models and the extraction of important features. Furthermore, a shapely additive explanation (SHAP) analysis was used to reveal the influence of the major factors and provide comprehensive explanations of their quantitative impacts on output. In addition, the XGBoost model was compared with the traditional logistic model and score systems commonly used in the ICU, including the Oxford Acute Severity of Illness Score (OASIS), sequential organ failure assessment (SOFA), and acute physiology score III (APS III). The present study would provide a reference for an XGBoost-based clinical decision support system to aid the early prediction of AKI in patients with GIB admitted to the ICU setting.

## Methods

### Data source

All data were extracted from the eICU-CRD (18) and MIMIC-IV version 1.0 databases (19). The MIMIC-IV contains comprehensive and high-quality data of 524,520 admissions (including 257,366 patients) admitted to intensive care units (ICUs) at the Beth Israel Deaconess Medical Center during 2008–2019. The eICU-CRD covered 200,859 ICU admissions (including 139,367 patients) between 2014 and 2015 at 208 hospitals in the United States. The research use of these databases was approved by the institutional review board of the Massachusetts Institute of Technology. All procedures were performed in accordance with the ethical standards of the Declaration of Helsinki and its later amendments or comparable ethical standards. We obtained permission to extract data from the MIMIC-IV database and eICU-CRD database.

### Cohort selection

GIB was defined according to the European Society of Gastrointestinal Endoscopy guideline (20, 21); AKI was diagnosed according to the KDIGO-AKI criteria based on serum creatinine in the first 48 h of ICU admission (22).

**Figure 1:**
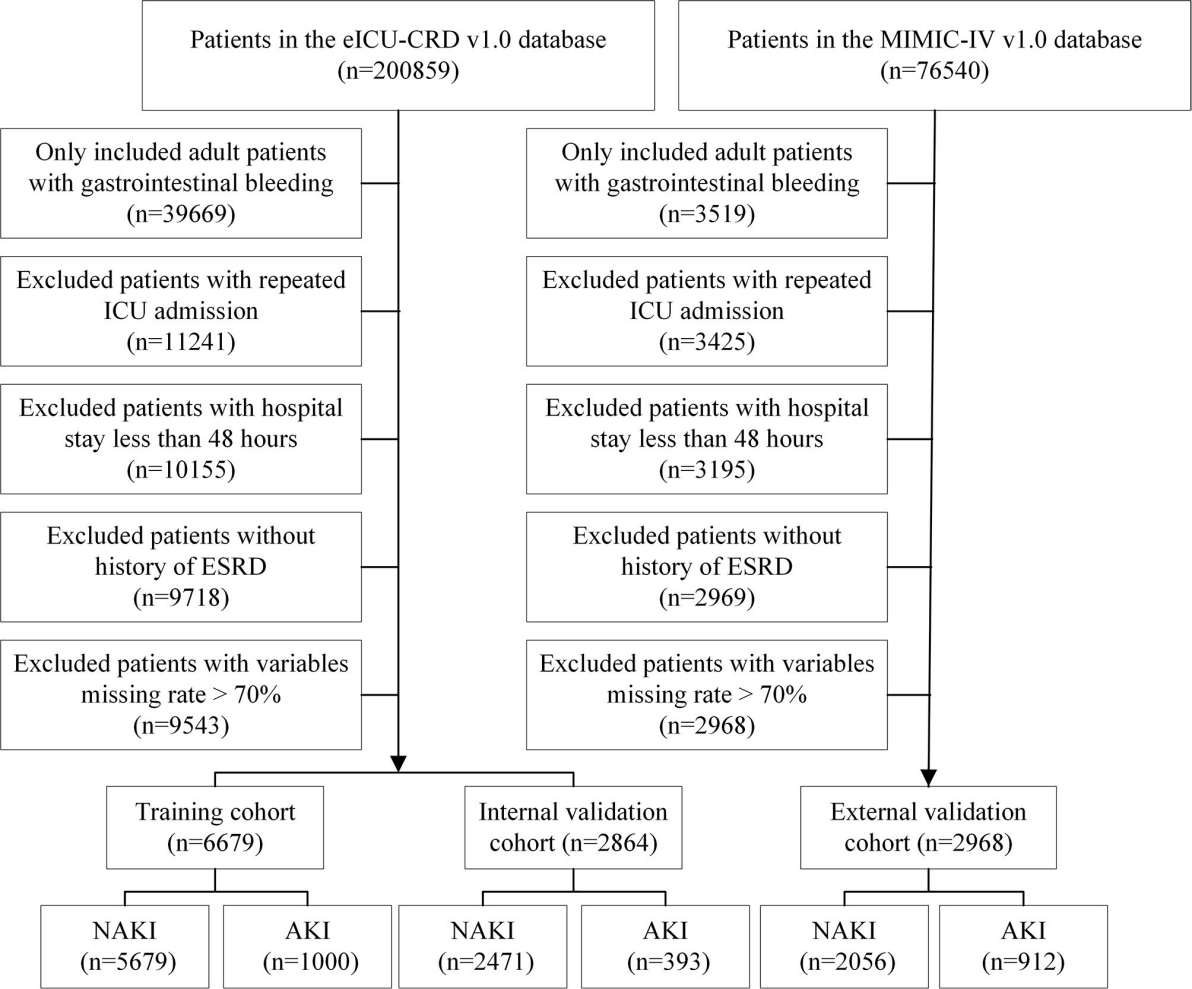
The flow chart of this study.

Patients with one of the following conditions were excluded: (1) an age of <18 years at first admission to the ICU, (2) a hospital stay of <48 h, (3) >70% of personal data missing, (4) repeated ICU admissions, and (5) a history of end-stage renal disease (ESRD). Finally, 6,679 patients with eICU-CRD and 2,968 patients with MIMIC-IV were included in this study. Moreover, patients from the eICU-CRD were randomly divided into training (*n* = 5,679) and internal validation cohorts (*n* = 1,000) at a ratio of 7:3. Patients from MIMIC-IV (*n* = 2,968) were used as an external validation set. A detailed flowchart is shown in Figure 1.

### Data collection and outcomes

Baseline characteristics and admission information, including age, sex, body mass index, bleeding site, comorbidities, severity score, and drug usage, were recorded. Initial vital signs and laboratory results were also measured during the first 24 h of ICU admission (19).

The primary outcome was AKI based on the KDIGO guidelines for serum creatinine within 48 h.

### Statistical analysis

For all continuous covariates, the mean values and standard deviations were reported, and categorical data were expressed as frequency (percentage). The chi-square test or Fisher's exact test was performed to compare differences between groups. Baseline characteristics were reported as training and validation cohorts. Baseline characteristics were compared using R software version 4.1.0. A *P*-value of < 0.05 was considered statistically significant. Modeling was performed using Python 3.6.4.

### AKI prediction model

**Figure 2:**
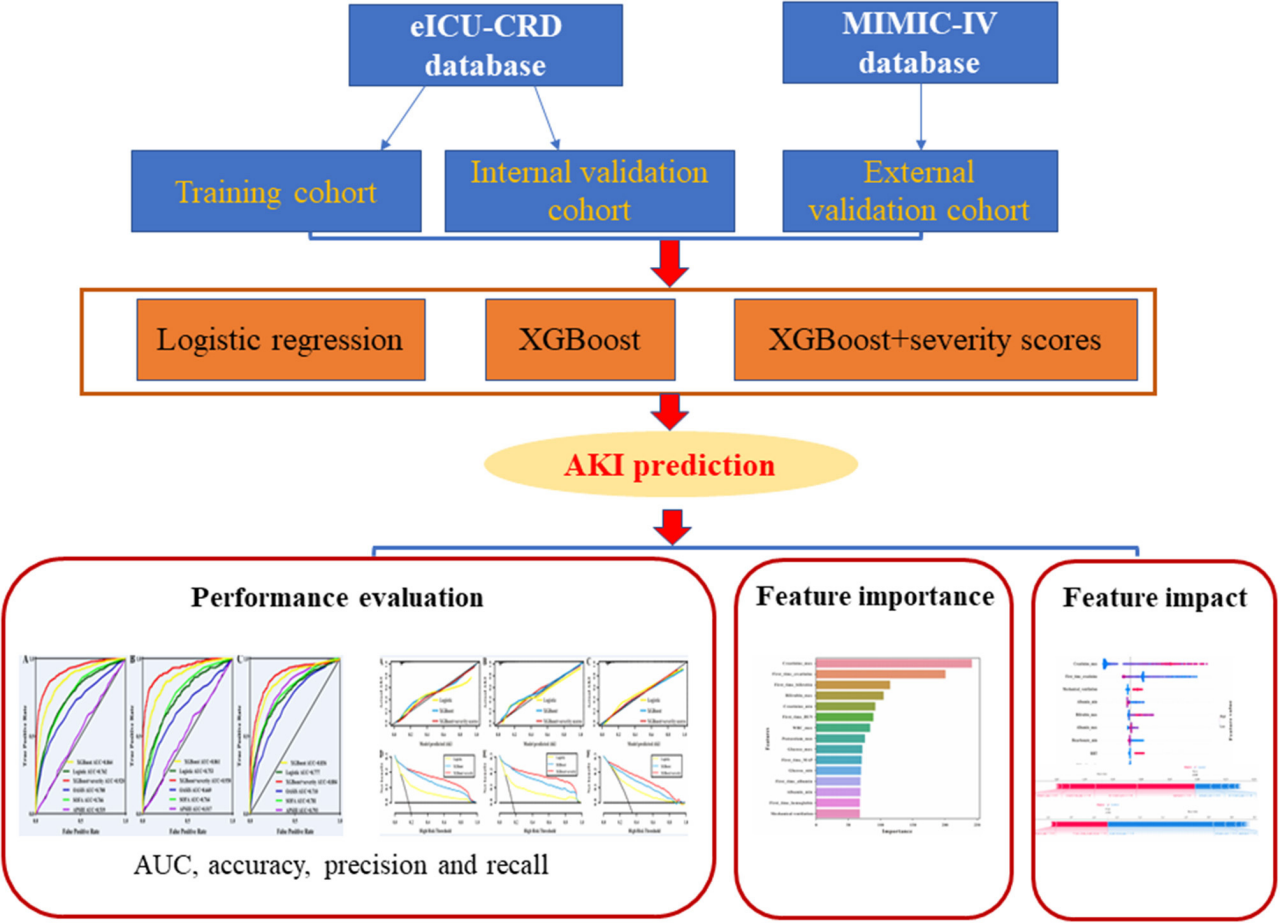
Framework of the prediction models.

Logistic regression, XGBoost, and XGBoost+severity scores (SOFA, OASIS, and APS III) were applied to build the prediction models. The XGBoost model was used as previously reported (23, 24). Moreover, all the machine-learning algorithms were implemented using the “sklearn” machine-learning library of Python programming software. The detailed XGBoost parameters are shown in Supplementary Table 1. The framework of the prediction models is illustrated in Figure 2.

### Performance evaluation

To assess and compare the predictive accuracy of XGBoost, XGBoost+severity scores, and logistic regression models, each model was assessed according to precision, recall, accuracy, F1 score, area under the receiver operating characteristic (AUC) curve, and area under precision–recall curve (AUC-PR) (25).

### Shapely additive explanation (SHAP) analysis

To further analyze the positive and negative effects of the important features identified for AKI prediction and investigate the relationship between them, a SHAP analysis was performed using Python 3.7.0. The SHAP value is the assigned predicted value for each feature of the data (26).

## Results

### Baseline characteristics

The incidence rate of AKI was 15.0% in the training cohort, 13.7% in the internal validation cohort, and 30.7% in the external validation cohort. Table 1 shows the baseline characteristics of all patients in the training, internal validation, and external validation cohorts, as classified by NAKI and AKI.

**Table 1:** Comparisons of baseline characteristics in all cohorts.

**Characteristics**	**Training cohort**			**Internal validation cohort**			**External validation cohort**		
	**NAKI**	**AKI**	**P-value**	**NAKI**	**AKI**	**P-value**	**NAKI**	**AKI**	**P-value**
*N*	5,679	1,000	-	2,471	393	-	2,056	912	-
Age, years old	67.0 ± 15.4	66.4 ± 15.0	0.260	67.2 ± 14.9	65.7 ± 14.5	0.065	65.7 ± 16.3	63.2 ± 15.7	< 0.001
Gender, male, *n* (%)	2,516 (44.3)	356 (35.6)	< 0.001	1,071 (43.3)	152 (38.7)	0.093	816 (39.7)	348 (38.0)	0.431
BMI, kg/m^2^	28.1 ± 7.9	28.7 ± 8.2	0.487	28.6 ± 7.6	29.1 ± 8.2	0.303	27.3 ± 8.1	28.7 ± 8.6	< 0.001
**Bleeding site**, ***n*** **(%)**			0.028			0.629			0.183
Upper	2,110 (37.2)	374 (37.4)		920 (37.2)	153 (38.9)		867 (42.2)	384 (42.1)	
Lower	1,398 (24.6)	210 (21.0)		613 (24.8)	89 (22.6)		475 (23.1)	236 (25.9)	
Unspecified	2,171 (38.2)	416 (41.6)		938 (38.0)	151 (38.4)		714 (34.7)	292 (32.0)	
**Interventions**, ***n*** **(%)**									
MV	1,400 (24.7)	539 (53.9)	< 0.001	656 (26.5)	220 (56.0)	< 0.001	626 (30.4)	585 (64.1)	< 0.001
RRT	104 (1.8)	165 (16.5)	< 0.001	34 (1.4)	68 (17.3)	< 0.001	29 (1.4)	167 (18.3)	< 0.001
Vasopressors	634 (11.2)	308 (30.8)	< 0.001	299 (12.1)	120 (30.5)	< 0.001	407 (19.8)	515 (56.5)	< 0.001
**Comorbidities**, ***n*** **(%)**									
Hypertension	3,014 (53.1)	548 (54.8)	0.329	1,350 (54.6)	213 (54.2)	0.915	667 (32.4)	200 (21.9)	< 0.001
Diabetes	1,540 (27.1)	321 (32.1)	0.001	653 (26.4)	128 (32.6)	0.013	607 (29.5)	287 (31.5)	0.306
Chronic kidney disease	599 (10.5)	252 (25.2)	< 0.001	267 (10.8)	96 (24.4)	< 0.001	420 (20.4)	239 (26.2)	< 0.001
Coronary artery disease	661 (11.6)	143 (14.3)	0.020	305 (12.3)	51 (13.0)	0.786	497 (24.2)	235 (25.8)	0.377
Congestive heart failure	782 (13.8)	188 (18.8)	< 0.001	349 (14.1)	65 (16.5)	0.235	561 (27.3)	308 (33.8)	< 0.001
Atrial fibrillation	854 (15.4)	146 (14.6)	0.757	339 (13.7)	55 (14.0)	0.945	506 (24.6)	265 (29.1)	0.012
Valvular disease	302 (5.3)	53 (5.3)	1.000	121 (4.9)	26 (6.6)	0.190	283 (13.8)	143 (15.7)	0.188
Arrhythmias	895 (15.8)	153 (15.3)	0.748	358 (14.5)	58 (14.8)	0.949	746 (36.3)	384 (42.1)	0.003
Liver disease	747 (13.2)	158 (15.8)	0.027	328 (13.3)	56 (14.2)	0.655	768 (37.4)	485 (53.2)	< 0.001
CCI, points	4.4 ± 0.7	4.8 ± 0.9	< 0.001	4.4 ±0.9	4.6 ±1.0	0.154	6.3 ± 2.9	6.9 ± 2.9	< 0.001
**Drugs usage**, ***n*** **(%)**									
ACEI/ARB	553 (9.7)	103 (10.3)	0.622	273 (11.0)	44 (11.2)	1.000	431 (21.0)	179 (19.6)	0.434
β blockers	1,724 (30.4)	354 (35.4)	0.002	763 (30.9)	141 (35.9)	0.055	1,145 (55.7)	557 (61.1)	0.007
CCB	406 (7.1)	89 (8.9)	0.060	171 (6.9)	35 (8.9)	0.190	237 (11.5)	120 (13.2)	0.231
Diuretic	1,660 (29.2)	423 (42.3)	< 0.001	773 (31.3)	150 (38.2)	0.008	1,057 (51.4)	691 (75.8)	< 0.001
Statin	775 (13.6)	154 (15.4)	0.153	378 (15.3)	57 (14.5)	0.754	703 (34.2)	289 (31.7)	0.196
Aspirin	778 (13.7)	202 (20.2)	< 0.001	358 (14.5)	90 (22.9)	< 0.001	595 (28.9)	316 (34.6)	0.002
PPI	3,750 (66.0)	652 (65.2)	0.634	1,644 (66.5)	254 (64.6)	0.495	1,963 (95.5)	886 (97.1)	0.041
**Score system, points**									
SOFA	3.2 ± 0.7	6.6 ± 1.9	< 0.001	3.3 ± 0.8	6.6 ± 1.0	< 0.001	4.8 ± 1.5	9.4 ± 2.8	< 0.001
OASIS	21.5 ± 9.1	29.1 ± 11.6	< 0.001	21.5 ± 9.0	28.9 ± 11.9	< 0.001	30.6 ± 8.5	37.6 ± 9.6	< 0.001
APSIII	35.7 ± 13.4	49.7 ± 12.7	< 0.001	35.8 ± 13.9	49.5 ± 14.5	< 0.001	46.6 ± 20.0	72.9 ± 28.2	< 0.001
**Laboratory values**									
MAP_first (mmHg)	80.6 ± 17.7	78.6 ± 20.0	0.001	81.0 (17.8)	77.9 (18.4)	0.001	82.3 ± 17.1	78.7 ± 18.9	< 0.001
MAP_min (mmHg)	61.8 ± 13.3	56.2 ± 14.2	< 0.001	62.1 ± 13.5	60.0 ± 14.7	< 0.001	59.5 ± 13.3	54.1 ± 13.6	< 0.001
MAP_max (mmHg)	108.8 ± 18.8	109.3 ± 21.2	0.410	108.0 ± 18.6	109.5 ± 21.6	0.135	103.5 ± 22.2	104.5 ± 29.3	0.358
WBC_first (10^9^/L)	11.7 ± 4.5	13.2 ± 4.9	< 0.001	11.8 ± 4.1	12.8 ± 5.4	0.220	10.5 ± 4.3	12.9 ± 5.7	< 0.001
WBC_min (10^9^/L)	9.7 ± 4.5	11.0 ± 4.8	< 0.001	9.7 ± 3.8	10.3 ± 4.2	0.168	8.9 ± 4.4	11.8 ± 4.6	< 0.001
WBC_max (10^9^/L)	12.9 ± 4.9	17.4 ± 5.8	< 0.001	13.0 ± 5.7	16.5 ± 6.6	< 0.001	12.3 ± 4.6	17.4 ± 5.0	< 0.001
HGB_first (mg/dL)	9.3 ± 2.9	9.7 ± 2.8	< 0.001	9.2 ± 2.8	10.0 ± 2.9	< 0.001	9.5 ± 2.2	9.8 ± 2.3	0.002
HGB_min (mg/dL)	8.1 ± 2.3	8.1 ± 2.3	0.991	8.1 ± 2.2	8.2 ± 2.4	0.155	8.5 ± 2.1	8.4 ± 2.1	0.092
HGB_max (mg/dL)	10.3 ± 2.2	10.9 ± 2.3	< 0.001	10.2 ± 2.1	11.0 ± 2.3	< 0.001	10.1 ± 2.0	10.3 ± 2.1	0.053
HCT_first (%)	28.4 ± 8.4	29.6 ± 8.4	< 0.001	28.1 ± 8.1	30.4 ± 8.6	< 0.001	28.8 ± 6.5	29.8 ± 6.9	< 0.001
HCT_min (%)	24.7 ± 6.7	24.7 ± 7.0	0.854	24.6 ± 6.5	25.1 ± 7.2	0.148	25.5 ± 5.8	25.2 ± 6.1	0.229
HCT_max (%)	31.2 ± 6.5	32.8 ± 6.9	< 0.001	30.8 ± 6.3	33.2 ± 6.9	< 0.001	31.1 ± 5.8	31.4 ± 6.2	0.219
PLT_first (10^9^/L)	220.6 ± 83.8	211.4 ± 70.8	0.032	218.7 ± 80.1	207.8 ± 78.7	0.096	191.3 ± 72.2	179.3 ± 71.8	0.013
PLT_min (10^9^/L)	176.5 ± 70.3	158.7 ± 73.4	< 0.001	177.9 ± 69.1	158.4 ± 67.0	< 0.001	168.4 ± 71.4	151.7 ± 72.0	< 0.001
PLT_max (10^9^/L)	225.3 ± 83.6	229.7 ± 88.9	0.312	224.2 ± 89.2	225.0 ± 89.4	0.904	214.4 ± 83.6	207.9 ± 88.1	0.256
Albumin_first (g/dL)	3.1 ± 0.6	2.7 ± 0.6	< 0.001	3.0 ± 0.7	2.7 ± 0.7	< 0.001	3.0 ± 0.6	2.9 ± 0.6	< 0.001
Albumin_min (g/dL)	2.7 ± 0.6	2.4 ± 0.5	< 0.001	2.7 ± 0.6	2.4 ± 0.7	< 0.001	3.0 ± 0.6	2.8 ± 0.7	< 0.001
Albumin_max (g/dL)	3.1 ± 0.7	2.8 ± 0.6	< 0.001	3.1 ± 0.7	2.8 ± 0.7	< 0.001	3.1 ± 0.6	3.0 ± 0.7	< 0.001
Bilirubin_first (mg/dL)	1.3 ± 0.5	2.4 ± 0.6	< 0.001	1.4 ± 0.5	2.8 ± 0.7	< 0.001	2.2 ± 0.8	5.2 ± 2.4	< 0.001
Bilirubin_min (mg/dL)	1.2 ± 0.4	2.2 ± 0.6	< 0.001	1.3 ± 0.4	2.6 ± 0.6	< 0.001	2.1 ± 0.9	5.5 ± 1.6	< 0.001
Bilirubin_max (mg/dL)	1.5 ± 0.5	2.9 ± 0.7	< 0.001	1.6 ± 0.5	3.3 ± 1.0	< 0.001	2.4 ± 0.9	6.4 ± 2.6	< 0.001
Bicarbonate_first (mEq/dL)	23.6 ± 5.0	22.4 ± 6.0	< 0.001	23.7 ± 4.7	22.4 ± 6.1	< 0.001	22.9 ± 4.6	21.5 ± 5.3	< 0.001
Bicarbonate_min (mEq/dL)	22.5 ± 4.8	19.4 ± 6.0	< 0.001	22.6 ± 4.6	19.6 ± 5.9	< 0.001	21.8 ± 4.7	19.1 ± 5.7	< 0.001
Bicarbonate_max (mEq/dL)	25.0 ± 4.4	24.7 ± 5.1	0.033	25.0 ± 4.2	24.8 ± 5.6	0.500	24.4 ± 4.2	23.0 ± 5.1	< 0.001
Anion gap_first, mEq/L	11.6 ± 5.0	13.1 ± 6.0	< 0.001	11.3 ± 4.1	12.9 ± 4.9	< 0.001	14.4 ± 4.7	16.3 ± 5.5	< 0.001
Anion gap_min, mEq/L	9.1 ± 3.7	10.2 ± 4.3	< 0.001	9.0 ± 3.6	10.0 ± 4.6	< 0.001	12.3 ± 3.2	14.3 ± 4.6	< 0.001
Anion gap_max, mEq/L	12.2 ± 5.0	15.2 ± 6.3	< 0.001	11.9 ± 4.9	14.7 ± 5.7	< 0.001	15.9 ± 5.4	19.1 ± 6.2	< 0.001
BUN_first (mg/dL)	35.7 ± 9.6	40.5 ± 9.8	< 0.001	35.4 ± 9.1	39.7 ± 10.7	0.005	31.6 ± 9.3	36.6 ± 9.5	< 0.001
BUN_min (mg/dL)	30.5 ± 9.2	35.5 ± 10.2	< 0.001	30.3 ± 8.8	33.9 ± 10.7	0.006	27.9 ± 9.5	39.4 ± 9.3	< 0.001
BUN_max (mg/dL)	37.2 ± 10.3	49.6 ± 12.2	< 0.001	36.6 ± 9.4	49.1 ± 14.5	< 0.001	33.3 ± 9.2	47.1 ± 9.7	< 0.001
SCr_first (mg/dL)	1.4 ± 0.3	2.3 ± 0.7	< 0.001	1.4 ± 0.4	2.3 ± 0.6	< 0.001	1.3 ± 0.4	1.9 ± 0.6	0.005
SCr_min (mg/dL)	1.2 ± 0.3	2.0 ± 0.6	< 0.001	1.2 ± 0.3	2.0 ± 0.5	< 0.001	1.1 ± 0.4	1.8 ± 0.5	< 0.001
SCr_max (mg/dL)	1.4 ± 0.4	2.8 ± 0.9	< 0.001	1.4 ± 0.4	2.8 ± 1.0	< 0.001	1.3 ± 0.3	2.2 ± 0.7	< 0.001
GLU_first (mg/dL)	152.9 ± 65.4	166.5 ± 68.7	< 0.001	153.9 ± 66.1	158.5 ± 68.2	0.422	139.1 ± 67.0	147.2 ± 69.1	0.009
GLU_min (mg/dL)	118.6 ± 45.5	114.4 ± 45.5	0.006	118.3 ± 34.4	115.8 ± 39.5	0.316	114.1 ± 39.2	117.6 ± 45.0	0.045
GLU_max (mg/dL)	164.3 ± 88.2	204.2 ± 93.8	< 0.001	164.6 ± 78.4	194.6 ± 93.5	< 0.001	166.7 ± 72.9	199.2 ± 73.0	< 0.001
Potassium_first (mmol/L)	4.2 ± 0.7	4.4 ± 0.9	< 0.001	4.2 ± 0.7	4.4 ± 1.0	< 0.001	4.2 ± 0.8	4.3 ± 0.9	< 0.001
Potassium_min (mmol/L)	3.8 ± 0.6	3.9 ± 0.7	0.867	3.9 ± 0.6	3.9 ± 0.7	0.763	3.9 ± 0.6	4.0 ± 0.7	< 0.001
Potassium_max (mmol/L)	4.4 ± 0.7	4.9 ± 0.9	< 0.001	4.3 ± 0.7	4.9 ± 1.0	< 0.001	4.5 ± 0.8	4.8 ± 1.0	< 0.001
Sodium_first (mmol/L)	137.8 ± 5.4	137.0 ± 6.4	< 0.001	138.0 ± 5.2	137.3 ± 6.4	0.015	138.6 ± 5.3	136.8 ± 6.6	< 0.001
Sodium_min (mmol/L)	137.3 ± 5.4	135.6 ± 6.1	< 0.001	137.4 ± 5.1	135.6 ± 6.2	< 0.001	137.3 ± 5.1	135.5 ± 6.2	< 0.001
Sodium_max (mmol/L)	140.1 ± 4.9	140.7 ± 6.2	0.002	140.2 ± 4.8	140.8 ± 6.2	0.020	140.3 ± 4.9	139.6 ± 6.1	0.001

NAKI, no acute kidney injury, MV, mechanical ventilation, RRT, renal replacement therapy, CCI, Charlson comorbidity index, ACEI/ARB, Angiotensin-converting enzyme inhibitors/Angiotensin receptor blockers, CCB, Calcium calcium blockers, PPI, proton pump inhibitor, SOFA, sequential organ failure assessment, OASIS, Oxford Acute Severity of Illness score, APSIII, acute physiology score III, MAP, mean arterial pressure, WBC, white blood cell, HGB, hemoglobin, HCT, hematocrit, PLT, platelets, BUN, blood urea nitrogen, SCr, serum creatinine, GLU, Glucose.

### Variable selection

**Figure 3:**
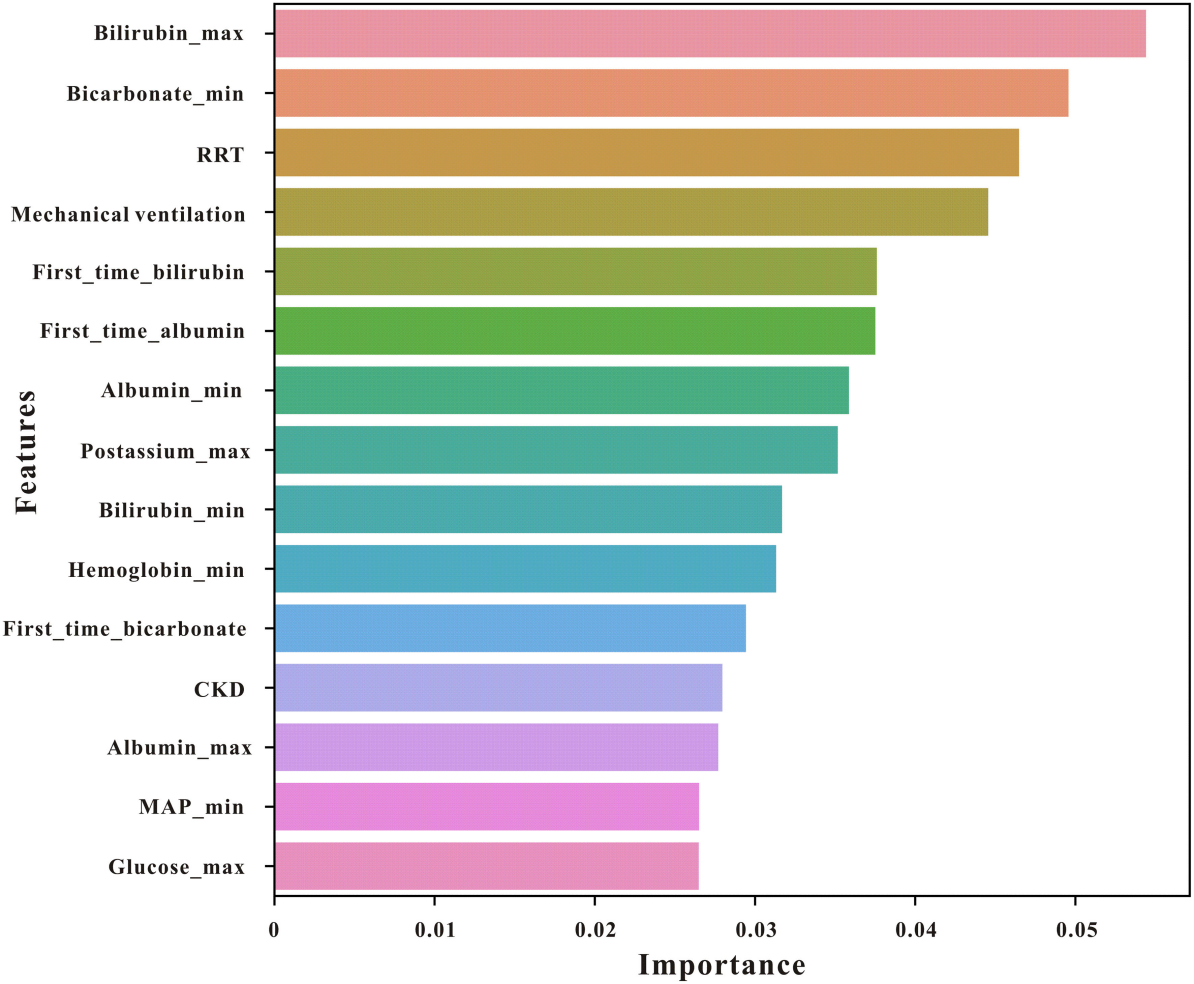
The top 15 features derived from the XGBoost model. Bilirubin_max, maximum serum bilirubin; bicarbonate_min, minimum bicarbonate; bilirubin_min, minimum bilirubin; RRT, renal replacement therapy; mechvent, mechanical ventilation; MAP, mean arterial pressure; CKD, chronic kidney disease.

The importance matrix plot for the XGBoost model is shown in Figure 3, revealing the top 15 most important variables that contribute to the model. Bilirubin (max) was the most important predictor variable for all prediction horizons, followed closely by bicarbonate (min), renal replacement therapy (RRT), mechanical ventilation, and bilirubin (first time).

### Model performance

**Figure 4:**
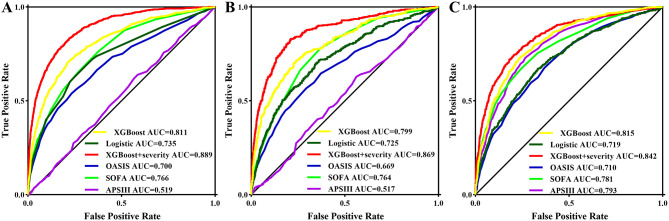
ROC curves of the prediction models using all features as well as three common severity scores for predicting AKI in the training set **(A)** and in the internal validation set **(B)** and in the external validation set **(C)**.

Three models, logistic regression, XGBoost, and XGBoost+severity scores, were used to predict AKI risk using all features. The accuracy, recall, precision, F1 score, AUC-PR, and AUC of XGBoost were higher than those of the logistic regression model. When XGBoost+severity scores (SOFA, OASIS, and APS III) were used, this model exhibited the best predictive ability compared to the XGBoost model only, as well as the logistic regression model with the highest accuracy, recall, precision, F1 score, AUC-PR, and AUC in the training cohort. The results in the internal and external validation cohorts were similar to the results in the training cohort (Table 2). Furthermore, ROC analysis was also performed to further check the performance of the three models, as shown in Figures 4A–C. The XGBoost+severity score model exhibited the largest AUC, followed by the XGBoost model, in all training, internal validation, and external validation cohorts.

**Table 2:** Performance of the prediction models using all features.

**Model**	**Accuracy**	**Recall**	**Precision**	**AUC**	**AUC-PR**	**F1 score**
**Training cohort**						
Logistic regression	0.80	0.62	0.69	0.74	0.68	0.78
XGBoost	0.84	0.70	0.82	0.81	0.75	0.82
XGBoost+severity scores	0.87	0.72	0.86	0.89	0.85	0.86
**Internal validation cohort**						
Logistic regression	0.82	0.64	0.63	0.73	0.64	0.79
XGBoost	0.87	0.75	0.82	0.80	0.72	0.83
XGBoost+severity scores	0.89	0.79	0.86	0.87	0.83	0.85
**External validation cohort**						
Logistic regression	0.71	0.69	0.68	0.72	0.65	0.70
XGBoost	0.76	0.76	0.70	0.82	0.72	0.75
XGBoost+severity scores	0.79	0.79	0.78	0.84	0.80	0.78

**Figure 5:**
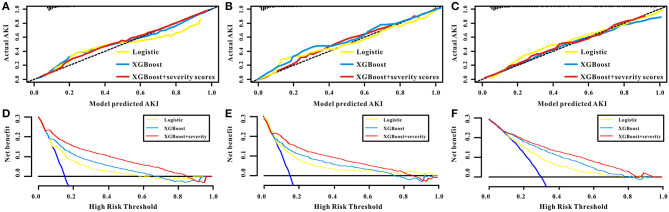
The performance of the models of logistic, XGBoost, and XGBoost+severity scores for AKI. The calibration curves of the logistic, XGBoost, and XGBoost+severity scores for AKI in the training set **(A)**, in the internal validation set **(B)**, and in the external validation set **(C)**. The decision curve analysis of the logistic, XGBoost, and XGBoost+severity scores for AKI in the training set **(D)**, in the internal validation set **(E)**, and in the external validation set **(F)**.

The calibration curves for the predictive models (logistic regression model, XGBoost model, and XGBoost+severity scores model) all showed high agreement between the actual probability and predicted probability in the training, internal validation, and external validation sets (Figures 5A–C). Subsequently, a decision curve analysis (DCA) was performed to determine the net benefit and clinical utility of the predictive models. The DCA curve also indicated that the three predictive models were all clinically useful and that the benefit of using the XGBoost+severity score model was superior to that of using the XGBoost and logistic regression models in all sets (Figures 5D–F).

### SHAP analysis

**Figure 6:**
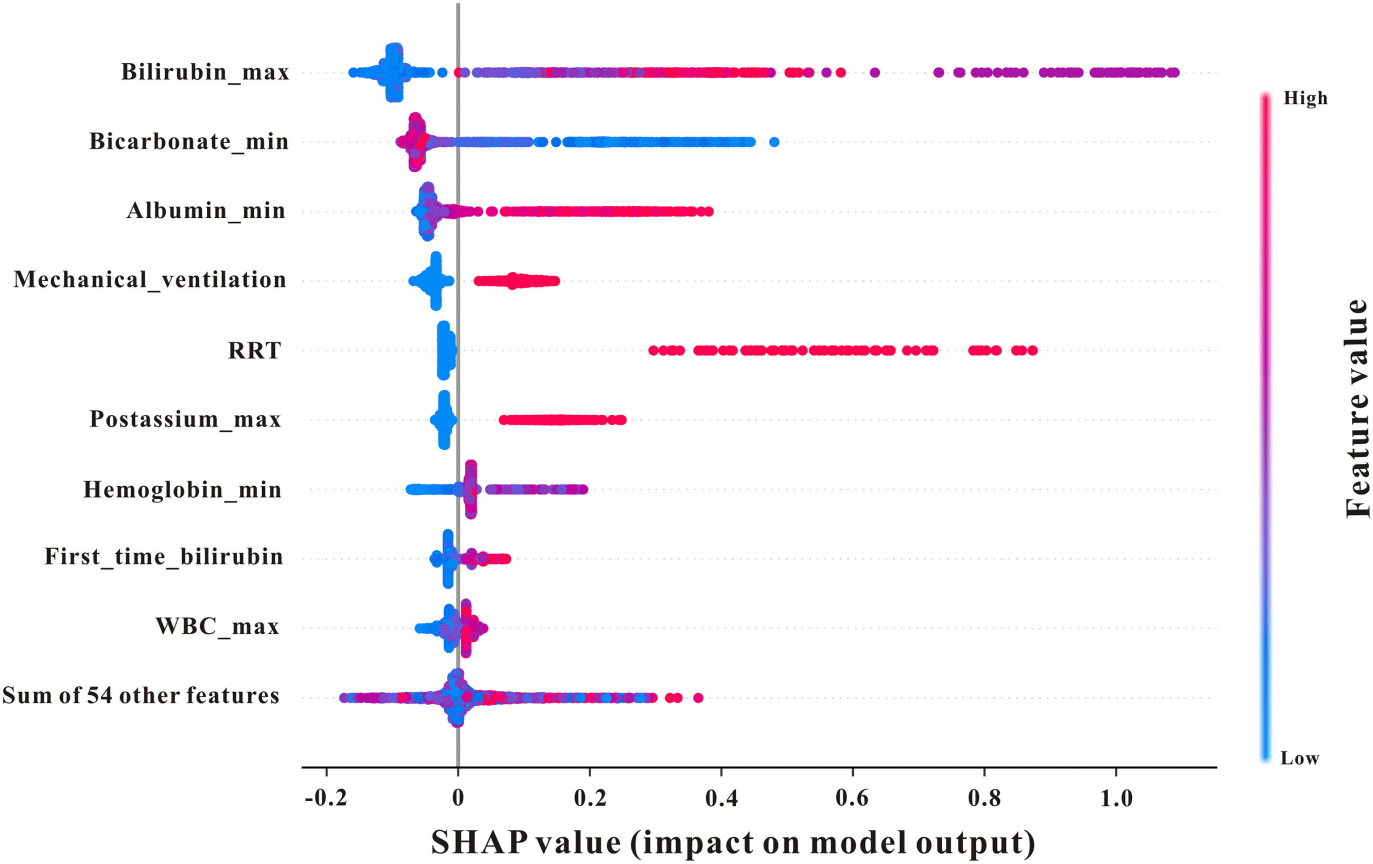
SHAP summary plot of the features of the XGBoost model. The higher the SHAP value of a feature, the higher the probability of AKI development. A dot is created for each feature attribution value for the model of each patient, and thus one patient is allocated one dot on the line for each feature. Dots are colored according to the values of features for the respective patient and accumulate vertically to depict density. Red represents higher feature values, and blue represents lower feature values. Creatinine_max, maximum serum creatinine; first_time_creatinine, the first measurement of serum creatinine after their ICU admission; bilirubin_min, minimum bilirubin; mechvent, mechanical ventilation.

To examine the influence of characteristics on the prediction results in more samples and analyze the similarities and differences in the important characteristics of patients with varying severities of AKI with different severities, a SHAP summary chart was used. As shown in Figure 6, bilirubin (max) ranked first in importance; the larger the bilirubin (max) in patients, the higher the probability of AKI development, suggesting that this indicator should be observed first in early prediction.

**Figure 7:**
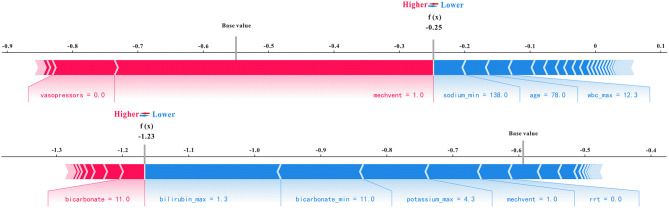
The two representative SHAP force plots of non-AKI and AKI patients in the training set.

Using all features as an example in the XGBoost model, which has excellent performance for predicting AKI, as well as the SHAP analysis method, representative non-AKI and AKI patients were selected to illustrate the effect of features on prediction ability. As shown in Figure 7, for predicting non-AKI patients, mechanical ventilation (mechvent) played a major positive role in prediction results, sodium (min) played a major negative role in predicting outcomes, and the SHAP value of the final model predicted for this patient was −0.25, which is < 0 and, therefore, considered to have successfully predicted the absence of AKI. For predicting AKI patients, the bicarbonate plays a major positive role in prediction results, the bilirubin (max) plays a major negative role in predicting outcomes, and the SHAP value of the final model predicted for this patient was 1.23, which is considered to have successfully predicted AKI.

## Discussion

Few studies have explored AKI-prediction models based on machine-learning techniques in critically ill GIB settings. The present study compared the predictive accuracy of the prediction of AKI in patients with GIB admitted to the ICU using the machine learning technique XGBoost, traditional statistical approach logistic regression analysis, and previous risk scoring models (SOFA, OASIS, and APS III). The results have shown that the XGBoost model had the largest AUC, accuracy, precision, and recall among all the techniques and risk scores. Moreover, the XGBoost+severity scores (SOFA, OASIS, and APS III) exhibited better AKI prediction performance than XGBoost. The XGBoost model-based prediction may induce a significant improvement in the prediction of AKI in patients with GIB admitted to the ICU. A risk estimator based on the XGBoost model was developed to determine the risk of AKI in high-risk patients with GIB.

Acute GIB is very common in patients in ICU (27). Mortality in patients with acute GIB is very high, approaching 48.5–65% (28, 29). According to previous reports, AKI occurs in ~25% of patients with acute GIB. Although AKI accounts for a small number of complications in critically ill patients with GIB, the mortality rate of critically ill patients with AKI is higher than that of patients with severe GIB without AKI. Xie et al. found that AKI occurred in 30% of patients with cirrhosis and that patients with cirrhosis and AKI had a worse prognosis (37 vs. 3%) (30). Moreover, a study by Kim et al. also showed that the 6-week mortality rate of cirrhotic patients with new-onset AKI was significantly higher than that of patients without AKI (31). The early identification of AKI can effectively prevent disease progression. However, there is currently a lack of reliable and effective predictive models for such patients, warranting researchers to develop a reliable AKI predictive model to identify high-risk critically ill patients with GIB.

With the advent of big data, ML has great potential in the field of AKI research owing to its unparalleled ability in data processing. Therefore, machine learning models may be powerful tools for AKI risk stratification and prediction (32). Several ML techniques have been used to predict AKI in different disease settings (33–35). However, the use of ML techniques to predict AKI in critically ill patients with GIB has not been investigated. As an ML technique, XGBoost is a highly efficient boosting ensemble learning model that originated in the decision tree model, using a tree classifier for better prediction results and higher operation efficiency (36). Several studies have found that XGBoost is superior to other machine learning techniques. Liu et al. reported that XGBoost exhibited the best performance in predicting mortality in patients with AKI in the ICU, with the highest AUC, F1 score, and accuracy compared with logistic regression, support vector machines, and random forest (37). Yue et al. aimed to establish and validate predictive models based on novel machine learning algorithms for AKI in critically ill patients with sepsis and found that the XGBoost model had the best predictive performance in terms of discrimination, calibration, and clinical application among all models, including logistic regression, SOFA, and the customized Simplified Acute Physiology Score (SAPS) II model (16). Qu et al. used support vector machine, random forest, classification and regression tree, and XGBoost models to predict AKI prediction, and compared to the predictive performance of the classic model using logistic regression, the results demonstrated that XGBoost performed best in predicting AKI among the machine learning models (34). Hence, the XGBoost algorithm was selected to structured and unstructured patient data from electronic medical records to develop an AKI prediction model in the present study. Consistent with previous reports (16, 34, 37), the XGBoost model was better than the traditional logistic regression model and previous risk scoring models (SOFA, OASIS, and APS III). The XGBoost+severity score model exhibited the highest accuracy, recall, precision, AUC, AUC-PR, and F1 score.

There are several studies in this respect. Although the studies have been conducted on different data and are not comparable, studies employed traditional ML techniques to predict AKI events, and XGBoost was the most commonly used algorithm. Using the MIMIC dataset for AKI prediction in the ICU setting, Zhang et al. (23) reported that XGBoost had a significantly greater ROC than the logistic regression model (0.86 vs. 0.728) in differentiating between volume-responsive and volume-unresponsive AKI. Zimmerman et al. (38) developed ML models to predict the new onset of AKI in critical care settings with a mean AUC of 0.783 by our all-feature, logistic-regression model. Sun et al. (39). used an ensemble learning algorithm for the early prediction of AKI with AUC 24 h ahead: 0.81, 48 h ahead: 0.78; MIMIC-III: AUC 24 h ahead: 0.95, and 48 h ahead: 0.95. In addition, Wang et al. (40) reported AUC above 0.83 with SVM as the best performer, and Qian et al. (41) reported that LightGBM had the best performance, with all evaluation indicators achieving the highest value (average AUC = 0.905, F1 = 0.897, recall = 0.836). Alfieri et al. (42) showed that AUC for deep learning is 0.907 and LR is 0.877. Shawwa et al. (43) indicated that a 30-feature model showed 0.690 in the Mayo Clinic cohort set and 0.656 in the MIMIC-III cohort. Because of different datasets (MIMIV-IV in our study and MIMIC-III in previous studies), comparing our results with other studies is difficult. However, in general, in comparison with the best results from previous studies, we also achieved a high AUC (XGBoost+severity scores: 0.89).

A SHAP analysis was used to determine the quantitative impact of each feature on AKI prediction based on SHAP values. The results of our study demonstrated that bilirubin and albumin were the most influential feature among all other physiological measurements A UK-wide study in acute medical units aimed to investigate patients who were at risk of developing AKI in hospitals and found that elevated serum bilirubin was independently associated with AKI development (44). Moreover, Wang et al. indicated that lower serum albumin levels were independently associated with a greater risk of contrast-induced AKI among patients who underwent percutaneous coronary intervention (45). Moreover, mechanical ventilation (mechvent), bicarbonate, and RRT also displayed strong predictive powers, which reflected their roles in AKI prediction in critically ill patients with GIB.

Nevertheless, this study has some limitations. First, the present study extracted data from two large public databases, and additional external clinical datasets may be needed to verify the results of this study. Second, we collected data during the first 24 h of ICU stay, and more dynamic time-point data are needed in future studies. Moreover, the variables we stated indicate that the predictive model's utilities are challenging, as they are at different time points (e.g., patients' first creatinine and highest bilirubin). Therefore, in reality, it would not be possible to use them to predict AKI risk until all time-point data were collected. Finally, the present study included an imbalanced dataset to check the performance of the machine learning and the predictive model developed using the machine learning algorithms could be biased and inaccurate. The results of this study should be further validated in the future.

## Conclusion

This study utilized an XGBoost-based model to predict AKI in patients with GIB admitted to the ICU. The results demonstrated that it is feasible to apply the XGBoost-based prediction models for the management of critically ill patients with GIB and that this model has better predictive performance than that of classic logistic regression methods and severity score models. The XGBoost-based model in this study has not been verified by an external cohort, and further studies are needed to determine the clinical application of the XGBoost-based model and to perform prospective and large sample experiments to verify our conclusion.

## Data availability statement

The raw data supporting the conclusions of this article will be made available by the authors, without undue reservation.

## Ethics statement

Research use of MIMIC-IV and eICU-CRD were approved by the Institutional Review Board (IRB) of the Massachusetts Institute of Technology (MIT). All procedures were performed according to the ethical standards of the Helsinki Declaration and its later amendments or comparable ethical standards. The studies were conducted in accordance with the local legislation and institutional requirements. Written informed consent for participation was not required from the participants or the participants' legal guardians/next of kin in accordance with the national legislation and institutional requirements.

## Author contributions

HS conceived and designed the study. YS extracted the data. HS and YS analyzed the data and drafted the manuscript. LL takes responsibility for the content of the manuscript including the data and analysis. All authors have approved the final version of the manuscript for submission and agree to be accountable for all aspects of the manuscript.
