# Supplementary material for: Early prediction of acute kidney injury in patients with gastrointestinal bleeding admitted to the intensive care unit based on extreme gradient boosting
Source: Front Med (Lausanne). 2023 Aug 31;10:1221602. doi: 10.3389/fmed.2023.1221602 (PMC10501398; doi:10.3389/fmed.2023.1221602)
Supplement: Supplementary file 1 [file Table_1.DOCX]

**Supplemental Table 1 Mathematical algorithm of XGBoost and logistic regression model**

| Algorithm | Parameters |
| --- | --- |
| XGBoost | XGBClassifier(base_score=0.5, booster='gbtree', colsample_bylevel=1, colsample_bynode=1, colsample_bytree=0.8, gamma=3, learning_rate=0.1, max_delta_step=0, max_depth=5, min_child_weight=1, missing=None, n_estimators=300, n_jobs=1, nthread=None, objective='binary:logistic', random_state=0, reg_alpha=0.1, reg_lambda=1, scale_pos_weight=1, seed=None, silent=None, subsample=0.8, verbosity=1) |
| Logistic regression | LogisticRegression(C=0.0001, class_weight=None, dual=False, fit_intercept=True, intercept_scaling=1, l1_ratio=None, max_iter=100, multi_class='warn', n_jobs= -1, penalty='l2', random_state=None, solver='warn', tol=0.0001, verbose=0, warm_start=False) |
